# Predictors of healthy ageing: public health policy targets

**DOI:** 10.1186/s12913-016-1520-5

**Published:** 2016-09-05

**Authors:** Agnieszka Sowa, Beata Tobiasz-Adamczyk, Roman Topór-Mądry, Andrea Poscia, Daniele Ignazio la Milia

**Affiliations:** 1 Department of Social Policy, Institute of Labour and Social Studies, Bellottiego 3B, Warsaw, Poland; 2 Department of Medical Sociology, Chair of Epidemiology and Preventive Medicine, Jagiellonian University Medical College, Grzegórzecka 20 St., 30-351 Crakow, Poland; 3 Department of Epidemiology and Population Health, Institute of Public Health, Jagiellonian University Medical College, Grzegórzecka 20 St., 30-351 Crakow, Poland; 4 Institute of Public Health, Università Cattolica del Sacro Cuore di Roma, Rome, Italy

**Keywords:** Healthy ageing, Healthy ageing predictors, Public health, Health policy

## Abstract

**Background:**

The public health policy agenda oriented towards healthy ageing becomes the highest priority for the European countries. The article discusses the healthy ageing concept and its possible determinants with an aim to identify behavioral patterns related to healthy ageing in selected European countries.

**Methods:**

The healthy ageing is assessed based on a composite indicator of self-assessed health, functional capabilities and life meaningfulness. The logistic regression models are used to assess the impact of the healthy lifestyle index, psycho-social index and socio-economic status on the probability of healthy ageing (i.e. being healthy at older age). The lifestyle and psychosocial indexes are created as a sum of behaviors that might be important for healthy ageing. Models are analyzed for three age groups of older people: 60–67, 68–79 and 80+ as well as for three groups of countries representing Western, Southern and Central-Eastern Europe.

**Results:**

The lifestyle index covering vigorous and moderate physical activity, consumption of vegetables and fruits, regular consumption of meals and adequate consumption of liquids is positively related to healthy ageing, increasing the likelihood of being healthy at older age with each of the items specified in the index. The score of the index is found to be significantly higher (on average by 1 point for men and 1.1 for women) for individuals ageing healthily. The psychosocial index covering employment, outdoor social participation, indoor activities and life satisfaction is also found to be significantly related to health increasing the likelihood of healthy ageing with each point of the index score. There is an educational gradient in healthy ageing in the population below the age of 68 and in Southern and Central-Eastern European countries. In Western European countries, income is positively related to healthy ageing for females.

**Conclusions:**

Stimulation physical activity and adequate nutrition are crucial domains for a well-defined public health policy oriented towards healthy ageing. The psychosocial elements related to social participation, engagement, networking and life satisfaction are also found to be health beneficial.

## Background

Increasing demographic pressure due to growing cohorts of older populations, results in changing priorities for the European countries. Political and social expectations influenced by the increasing life expectancy are expressed in the concept of ageing with high quality of life, lower level of morbidity, fewer years of disability and high life standards [1]. This new approach to the older stage of life has been associated with an increasing research and public interest in developing an innovative, but still not so well defined, multifaceted concept of healthy ageing (interchangeably named by some researches as “active aging” or “successful ageing”), especially in relation to the main priorities for the public health policies in the ageing era. This study focuses on the relation between healthy ageing (i.e. being healthy at older age) and three groups of factors: healthy lifestyle, psychosocial factors and socio-demographics.

### Defining healthy ageing

Health promotion addressed to seniors found a place in Europe's health programs under the heading healthy aging (particularly within the Second Program of Community Action in the Field of Health 2008–2013 “Together for Health”). There are many definitions of healthy aging – a concept similar to successful and active which all aim at ageing in good health and well-being. Cosco [2] after a systematic review of the existing literature and research, noticed 105 operational definitions of successful and healthy ageing and found that 92.4 % of them included physiological constructs (e.g. physical functioning), 49.5 % engagement constructs (e.g. involvement in voluntary work), 48.6 % well-being constructs (e.g. life satisfaction), 25.7 % personal resources (e.g. resilience), and 5.7 % extrinsic factors (e.g. finances) [2]. While many definitions of healthy ageing have been developed in the last decades, there is no consensual agreement on its content, what is a fundamental weakness of the concept and what might be problematic for the creation of a comprehensive public health policy [2, 3]. Definitions used usually have been based on one of the two different theoretical perspectives. The first one refers to the bio-medical model of ageing, with the stress on physical health, functional and cognitive capacity, eventually supported by the psychological dimension and social activity. The second perspective, independently from physical health, is focused on the psychosocial dimensions of healthy ageing: psychological well-being and meaningful social activities performed by older people, social participation in different social networks, as well as on seeking some new opportunities to enjoy a good quality of life in older age.

The classical definition of ageing in good health, referring to the concept of successful ageing, created by Rowe and Khan is based on the balance between the three components: absence of disease and disease-related disability, high functional capacity and active engagement with life [4, 5]. It significantly influenced several other, later definitions. At first, most of them were based on bio-medical approach to healthy ageing using objective, very often clinical, indicators while later subjective indicators of health status such as self-rated health have been additionally applied in research. Presented definitions began to be very precise in assessing the criteria which should be met to define the healthy ageing such as absence of any important or specific illness like cardiovascular disease, chronic obstructive pulmonary disease or previous cancer diagnosis; no limitations in performing basic Activities of Daily Living (ADL); freedom from clinically significant cognitive impairments and depression symptoms; high cognitive and physical functions and active life engagement [6–12]. These measures were often supported by specific indicators of clinical assessment [13].

The psychosocial perspective on ageing in good health encompasses multidimensional models showing the process of adaptation to older stage of life based on objective and subjective indicators as well as on lay people definitions of healthy ageing [14]. Psychosocial models of healthy ageing refer to the concept of “doing and having” as well as “going and doing” [15], and include experiences related to continuation of meaningful previous social activities (having paid work, volunteer work, domestic tasks, active participation in community, social support from family, friendship, economic security, sport, travel and creative activities). Special attention has been paid to self-assessment of heath, health behaviors, mental health; social resources such as social networks, social support, social participation, feeling of social belongings, satisfaction, happiness and enjoying life, acceptance of age, independence, autonomy, empowerment [16–23].

The World Health Organization (WHO) has undertaken efforts to operationalize the healthy ageing definition for the purpose of constructing well-defined public health actions. WHO proposes to define healthy ageing as “*the process of developing and maintaining the functional ability that enables well-being in older age”* [1]. The healthy ageing comprises of functional abilities, intrinsic capacity being the outcome of physical and mental abilities of older people, impact of physical and social environment and well-being. This multidimensional construct is synonymous to a definition of good quality of life in older age perceived as “as an individual’s perception of his/her position in life in the context of the culture and value systems in which he/she live and in relation to goals, expectations standards and concerns” [24].

### Healthy ageing predictors

The multifaceted phenomenon of healthy ageing can be attributed on the one hand to the overall life experiences that can impact the health status in older age and to the experiences related to the later stage of life on the other hand. The life-course approach attempts to explain the role of possible effects of life experiences on the health status and healthy ageing, especially the accumulation of advantages and disadvantages over the life span and latency effect of social experiences acquired in early life on adult and older age health status, including the effect of social conditions in early life on acquired social status and eventually on health in adulthood [25–28]. This perspective focuses on different factors with life chances and life choices that might significantly influence health status, lifestyle as well as health and social inequalities in older age. Health inequalities in older age as a special subject of research have recently been developed, but for years, research on this topic has concentrated mostly on adult, but not older population [27]. Although the research of the last decade provides evidence of inequalities in longevity and time spent in disability between social, occupational and economic groups, some data show that inequalities in health in older age tend to be smaller than among adult population and tend to decrease with increasing age [27, 29].

Among different health status predictors at older age, it is necessary to stress social status, education, wealth and income [30–33]; retirement in relation to the lost locus of control and effort-reward mechanisms [25, 34]; lifestyle indicated by nutrition, physical activity, smoking and alcohol consumption [27, 35–42], as well as specific psycho-social factors like social networks, social support and social participation [27, 43, 44].

The gradient in health status by education and income has been confirmed in various studies, pointing to the positive impact of education and wealth/income on individual health in adulthood in European countries [45–47]. Seniors with higher education tend to be in better health, more frequently report higher life satisfaction, interest with life and well-being, and are more socially engaged [27, 30, 45]. Social status, income and education influence lifestyle choices that are important for healthy ageing [33, 45] as well as increase options for dealing with ill health by better opportunities for the health care use and better quality of care [27] although the relation between social position and health behaviors in older age might be less clear due to premature mortality, change of habits related to occurrence of various illnesses or cohort effects [27].

A crucial element for healthy ageing is healthy lifestyle. Individuals living healthy not only survive longer, but live longer in better health with occurrence of disability and age related diseases postponed to the last years of life [38]. Healthy behaviors are also found to support recovery process in case of illness [39, 40]. Most importantly, these relations are found to be important even for the population above the age of 75 pointing to the need for promotion of healthy lifestyle also among the oldest population [40]. Various studies point that lifestyle factors such as smoking, unhealthy dietary patterns and obesity, physical inactivity and sedentary lifestyle as well as heavy drinking are associated with the onset of mobility limitations [38–40]. Inadequate nutritional habits that lead either to malnutrition or obesity are related to the onset of morbidity, physical disability, poor quality of life and – eventually – mortality. Adequate nutrition and physical exercises are the actual cornerstones of interventions to prevent and contrast non-communicable diseases, frailty and sarcopenia [48]. Other types of behaviors are also of great importance. Myint et al. [41] estimated quantitatively the negative health effect of smoking as an equivalent to being 7 years older and negative health effect of physical inactivity as equivalent of being 13 years older. There is evidence that cessation of smoking at the age of 65–74 immediately decreases the risk of mortality due to coronary heart disease and other diseases, including pulmonary diseases where the risk declines within the following five years [35]. Physical activity is found to decrease the risk of cardiovascular system diseases, prevent osteoporosis and improve mental health. Even moderate leisure time physical activity is shown to have protective effects against dementia, developing depression symptoms and mobility limitations among the older cohorts [36, 44, 49, 50].

Social networks are complementary and important element of healthy ageing and might be a stimulus for undertaking a healthy lifestyle. Social engagement, a potential for support or satisfaction with support and social networks are shown to have protective effect and prevent illness, mediate the negative effect of illness or mediate the relation between health status and socio-economic status [27, 51, 52]. Social networks are an important predictor of mortality and occurrence of disease, especially mental illnesses [43, 53–55]. Berkman [43] points to the more significant impact of the closest, family ties on reducing the risk of mortality. Other studies [53, 54] show the importance of relations with friends and other types of social involvement for the decrease in the risk of morbidity and mortality. Loneliness and poor social support are found to have a strong association with mental illnesses, especially depression, and may solely be an independent predictor for depression symptoms in older population [56–58]. They are linked with higher blood pressure, worse sleep and worse cognition in older people [56] as well as with functional limitations among women [58]. Social participation and social capital are other factors that are shown to impact health status. Participation in social activities, voluntary work or religious practices might decrease the risk of morbidity, functional limitations and mental illness and are related to the lower mortality risk. Participation in voluntary activities is found to be positively related to an improvement of physical health, reduction of functional decline, reduction of depression and improvement of psychological wellbeing [59–61]. There is also evidence of the impact of religious involvement, especially in the religious activities of a public character, on adult mortality risks [62–64].

### Healthy ageing as a public health policy target

European countries vary in health status in older age [65] and these differences together with age specifics should be accounted for when designing adequate public health policy and health promotion. The WHO agenda on ageing [1] points that public health policy should take into account the health diversity in older age related to the decline of capacities. Moreover, activities should be designed responding to the needs the specific age cohorts. Most of the health policy actions concentrate on the prevention of non-communicable diseases and maintaining functional capacity [1]. At the same time, key health behaviors such as smoking cessation, physical activity and adequate nutrition are among the factors that contribute not only to reducing the risk of non-communicable disease in adulthood, but also to the greater capacity for good self-perception and healthy ageing. Public health policy tackling lifestyle of older people is found to be among the central strategies that might reverse or delay frailty and decline in physical and cognitive functioning [1]. However, cultural and environmental factors might be of great importance for undertaking healthy behaviors in older age and there is a great differentiation of the prevalence of healthy habits among older population in European countries.

Presented analysis aims at the identification of healthy ageing predictors, with special attention given to healthy lifestyle and psychosocial factors that could be health policy targets, accounting for differences in the prevalence of good health status and different composition of predictors of healthy ageing in selected European countries, by sex and age.

## Methods

The analysis is performed based on the Survey of Health, Ageing and Retirement in Europe (SHARE) wave 4 (2010–2011). The SHARE is a multidisciplinary and cross-national panel database of micro data on health, socio-economic status as well as social and family networks of more than 80,000 individuals from 20 European countries (+Israel) aged 50 or over. There had already been 5 waves of the study that run from 2004 to 2015. The SHARE main questionnaire consists of 20 modules on health, socio-economics and social networks. All data are collected by face-to-face computer-aided personal interviews (CAPI). The databases of the SHARE are publicly accessible for research use, and are the only databases with such access from the large studies including all dimensions of health and health determinants. The wave 4th is the newest full survey from the study (Wave 5- SHARELIFE has other objectives). The methodology, compliance and databases were described previously [66–68]. In this study, the data from 6 selected European countries are used. These countries represent different welfare policies, traditions and behavioral patterns: Netherlands and Germany represent Western Europe, Italy and Spain represent Southern Europe, Poland and Hungary represent Eastern -Central Europe. The analysis covers 5139 men and 5909 women from the six selected countries. The data are representative for the respective country populations.

### Healthy ageing assessment

The dependent variable of healthy ageing is constructed as a binary variable with a reference to three dimensions that have been identified in the overview of healthy ageing definitions: health status self-assessment, functional capacity and assessment of the perceived meaning of life. The healthy ageing is assessed as reporting good or better than good health status (self-assessed health – SAH), having no limitations in the Activities of Daily Living (ADL) and reporting that at least sometimes (sometimes or often) life has a meaning. The list of ADL included the following items: walking 100 m; sitting for two hours; getting up from a chair after sitting for a long period; climbing several flights of stairs without resting; climbing one flight of stairs without resting; stooping, kneeling, or crouching; reaching or extending arms above shoulder level; pulling or pushing large objects, such as a living room chair; lifting or carrying weights over 10 lb (5 kg), such as a heavy bag of groceries; and picking up a small coin from the table. The self-perceived meaning of life is a measure of psychological capabilities. Earlier studies point that the will to live and the perceived meaningfulness of life are associated with the longevity of older adults and their well-being [69].

The healthy ageing indicator composed of the three above listed measures is well rooted in previous (see above) research. Most of the healthy ageing definitions refer to some sort of subjective health measure, functional or independence assessment and psychological construct. Several definitions use healthy ageing measures similar to the indicator presented in this study. Castro-Lieonard [70] used an indicator based on two components: well-being and self-assessed health, Cerning [71] created an indicator of physical functioning, cognitive functioning and self-assessed health, Lopez et al. [72] used physical functioning, life satisfaction and self-assessed health together with cognitive functioning and activity, Dionigi et al. [73] used an indicator including functional independence, happiness and self-assessed health together with social engagement.

### Healthy lifestyle and psychosocial indexes

The behavioral analysis uses two indexes of healthy lifestyle indicators and psychosocial indicators. The healthy lifestyle indicator refers to the domains of smoking, physical activity (moderate or vigorous and nutrition (consumption of fruits and vegetables per week, drinks per day and regular consumption of more than 3 meals per day). Each domain was valued with a score from 0 to 2, depending on its potential health effect as identified in the literature. The index follows the WHO guidelines on the selected domains [74, 75], though in a simple manner as the SHARE database provides only information on the types and frequencies of selected behaviors, not accounting for their quality (i.e. the calories intake of specific types of food, time spend on specific physical activities). The index was created as a sum of the points for each of the domains (Table 1).

**Table 1:** Characteristics of the sample by sex

	Categories	Males		Females		*Description of indexes – no. of points attributed*	Index
Variables		*N*	%	*N*	%		
Age groups	60–67	1976	38.5	2324	39.3		
	68–79	2399	46.7	2546	43.1		
	> = 80	764	14.9	1039	17.6		
Education	Elementary	2529	51.2	3782	65.4		
	Secondary	1535	31.1	1394	24.1		
	University	876	17.7	609	10.5		
Country group	Western Europe	1445	28.1	1589	26.9		
	Southern Europe	2301	44.8	2598	44		
	Central-Eastern Europe	1393	27.1	1722	29.1		
Healthy ageing	Worse than good SAH. functional limitations. perception that life has no meaning	2717	52.9	3484	59		
	At least good SAH. no functional limitations. perception of life meaningfulness	2422	47.1	2425	41		
Current job situation	Non-working (retired. unemployed. homemaker. permanently sick or disabled. rentier. student. etc.)	4632	91.2	5594	95.7	0	Psychosocial index
	Working (employed or self-employed)	449	8.8	253	4.3	1	
Activity in last 12 months	No activities	1284	25.5	1504	25.9	0	
	Activities at home (read books. magazines. did word or number games. chess. cards)	1773	35.2	1916	33	1	
	Activities outside of house (voluntary or charity work. educational course. sport. social club. taking part in religious. community organizations.	1982	39.3	2379	41	2	
Network satisfaction (scale from 0 to 10 where 0 means completely dissatisfied and 10 means completely satisfied)	0–5 points	43	0.9	48	0.9	0	
	6–10 points	4728	99.1	5479	99.1	1	
Life satisfaction (scale from 0 to 10 where 0 means completely dissatisfied and 10 means completely satisfied)	0–5 points	778	15.2	1170	19.9	0	
	6–10 points	4329	84.8	4721	80.1	1	
Smoking status	Current smoking	922	21.2	589	14.2	0	Life style index
	No smoking currently	3428	78.8	3573	85.8	1	
Vigorous physical activity	Less than once a week	2979	58.5	3956	67.7	0	
	Once a week	611	12	619	10.6	1	
	More than once a week sports or activities that are vigorous	1498	29.4	1271	21.7	2	
Moderate physical activity	Once a week or less	1836	36.1	2544	43.5	0	
	More than once a week activities requiring a moderate level of energy	3252	63.9	3302	56.5	1	
Vegetables consumption	Less than 3–6 times a week	411	8	355	6.1	0	
	3–6 times a week	903	17.6	888	15.2	1	
	Every day serving of fruits or vegetables	3776	73.5	4604	78.7	2	
Drinks consumption	6 cups of more a day drinks of tea. coffee. water. milk. fruit. soft drinks	374	7.3	439	7.5	0	
	3–5 cups	1534	30.1	1989	34	1	
	1–2 cups	3183	62.5	3418	58.5	2	
Regular meals	Meals not regular	569	11.1	595	10.1	0	
	Regular meals	4570	88.9	5314	89.9	1	
		N	mean (SD)	N	mean (SD)		
Lifestyle index		4717	6.26 (1.70)	5475	6.20 (1.65)		
Psychosocial index		4347	3.12 (0.98)	4156	3.03 (0.99)		

Source: own calculations

The psychosocial index includes domains of participation in the labor market (employment or self-employment), participation in organized activities of social character (volunteering, learning, sports, clubs, religious and community organizations), undertaking leisure activities at home (reading, playing games or chess, doing crosswords), satisfaction from the social network and life satisfaction. Again, a coding was applied to each type of activity (from 0 to 2 points) and the index was created as a sum of the points for each dimension, separately for the lifestyle and psychosocial index (Table 1). For the index calculation, only the data is used where the status of all components was known.

The descriptive statistics include the frequency distribution for all categorical variables as well as the mean and standard deviation (SD) for the continuous variables. For comparing of the health status for country and education groups, chi square test was used and for lifestyle and psychological indexes, t-test was used (normal distribution of indexes was estimated based on skewness parameter). The multivariable analysis of association between health and potential predictors analysis used a logistic regression model with health status as a dependent variable and a set of independent explanatory variables. The core independent variables are the healthy lifestyle index and psychosocial index based on the behaviors that have been identified in the literature as important for the healthy ageing process (see above). As confounding variables education (all models), income (log transformed), age group, country group (where applicable) are used. Three age groups have been distinguished: 60–67 years of age, 68–79 years of age and the group above 80 years of age. The grouping is based on the assumption that behaviors and health needs of older population might change depending on their involvement in the outside activities, especially labor market. The first group includes people that are still potentially labor market active. The upper age limit for this group is equal to the retirement age which is foreseen to increase to 67 years of age in most of the analyzed countries in the years to come. In Germany, the retirement age is gradually increasing from 65 to 67 by 2027; in the Netherlands, it is increasing from 65 to 67 by 2024; in Italy, it equals 66 years of age for men and 64 for women; in Spain, it is gradually increasing from 65 to 67 by 2027; in Poland, it is gradually increasing from 65 to 67 by 2020 for men and from 60 to 67 by 2040 for women; in Hungary, it is gradually increasing from 62 to 65 by 2022. The second age group (68–79) is represented by people who have reached retirement age, but still have a great potential of active ageing and involvement in social activities. The third group consists of the oldest old (80+), for whom the potential of activity is lower while care needs increase in line with health deterioration. Education is assessed based on the ISCED-97 scale as a set of binary variables of primary, secondary and higher education. Financial situation is assessed based on the reported income (continuous variable). Regression models are run separately for the three age groups: 60-67/68-79/80+ and for the above mentioned three groups of countries. All analyses are carried out separately for men and women. The analysis is done using SPSS v. 23, for the statistical significance *p* < 0,05 is used.

## Results

There is a great disproportion of older people (60+) reporting healthy ageing (i.e. being healthy) across countries. In the countries of Western Europe (Netherlands, Germany), almost 60 % of older people (60+) report being in good health, having no functional limitations and finding a meaning in life. In Western Europe, not only older people have higher propensity for healthy ageing, but there are almost no differences observable between sexes. In the countries of Southern Europe, the share of individuals reporting healthy ageing is lower and the differences between men and women are large (accounting to 10 pp.) with 48 % of men and 38 % of females reporting healthy ageing. In the countries of Central-Eastern Europe, the picture is quite different with only 33 % of men and 29 % of women aged 60+ reporting healthy ageing (Table 2).

**Table 2:** Percentage of person with good health status by country, education groups and sex

Items:		Males		Females	
		*N*	%	*N*	%
Country group	Western Europe	849	58.8 %	937	59.0 %
	Southern Europe	1109	48.2 %	986	38.0 %
	Central-Eastern Europe	464	33.3 %	502	29.2 %
	*p* value	<0.001		<0.001	
	(difference between health and country groups				
Education	Primary	1057	41.8 %	1313	34.7 %
	Secondary	738	48.1 %	693	49.7 %
	University	534	61.0 %	365	59.9 %
	*p* value	<0.001		<0.001	
	(difference between health and education groups				

Source: own calculations

On average in the six selected European countries, men are found to have higher propensity for healthy ageing (i.e. being healthy) than women as 47 % of men and 41 % of women respond positively to health-related items that contribute to healthy ageing. There are however no sound sex differences in the lifestyle undertaken by older men and women that might be of importance for healthy ageing. On average, men have the score of the healthy lifestyle index at the level of 6.26 and women at the level of 6.2 out of the maximum of 9 points. The average level of the psychosocial index accounts to 3.12 for men and 3.03 for women out of the maximum of 5 points (Table 1). At the same time both indexes are found to be related to healthy ageing. The average score of the lifestyle index is higher on average by 1 point for men and 1.1 point for women ageing healthy (i.e. being healthy) than for men and women with poorer health. Also the average score of the psychosocial index is higher for individuals with propensity to better health – on average by 0.6 point for men and by 0.7 for women (Table 3).

**Table 3:** Mean (SD) of psychosocial and lifestyle index value in groups of health and by sex

		Males		Females	
Index	Items	*N*	Mean (SD)	*N*	Mean (SD)
Lifestyle index	Health status good	2076	6.83 (1.6)	1837	6.79 (1.6)
	Health status not good	2271	5.73 (1.6)	2319	5.72 (1.6)
	*p* value	<0.001		<0.001	
	(Difference between health groups				
Psychosocial index	Health status good	2301	3.42 (0.9)	2317	3.41 (0.9)
	Health status not good	2416	2.83 (1.0)	3158	2.76 (1.0)
	*p* value	<0.001		<0.001	
	(Difference between health groups				

Source: own calculations

The results of the multidimensional analysis of healthy ageing predictors in different age groups point to the importance of both indexes: lifestyle and psychosocial (Table 4). The odd ratios of being healthy in older age increase with each point of the lifestyle index by 32.5 % for males and 18.7 % for females aged 60–67; 32.6 % for males and 28 % for females aged 68–79 and 53.5 % for males and 36.9 % for females aged 80+. Social participation, networking and life satisfaction are also of great importance, with the odds ratios of healthy ageing increasing with each point of the psychosocial index by 55.2 % for males and 61.3 % for females aged 60–67; 73.5 % for males and 71.9 % for females aged 68–79 and 76.2 % for males and 45.4 % for males aged 80 +.

**Table 4:** Multidimensional analysis of health ageing predictors by age groups – logistic regression results

Item		Age groups											
		60–67				68–79				> = 80			
Male or female		OR	95 % CI			OR	95 % CI			OR	95 % CI		
			Lower level	Upper level	*p*		Lower level	Upper level	*p*		Lower level	Upper level	*p*
Male	Income (log.)	0.957	0.866	1.059	.396	1.082	0.958	1.222	0.204	0.981	0.736	1.308	0.896
	Elementary educ.	1				1				1			
	Secondary educ.	1.664	1.242	2.229	0.001	1.020	0.784	1.327	0.883	0.617	0.341	1.116	0.110
	University educ.	2.214	1.546	3.172	0.000	1.359	0.985	1.875	0.062	0.660	0.347	1.252	0.203
	Index lifestyle	1.325	1.232	1.424	0.000	1.326	1.242	1.417	0.000	1.824	1.533	2.170	0.000
	Index psychosocial	1.552	1.362	1.767	0.000	1.735	1.531	1.967	0.000	1.762	1.366	2.273	0.000
	Central Europe	1				1				1			
	Western Europe	2.259	1.635	3.119	0.000	1.532	1.115	2.106	0.009	1.440	0.721	2.878	0.302
	Southern Europe	2.969	2.149	4.102	0.000	2.376	1.753	3.220	0.000	1.485	0.770	2.867	0.238
Female	Income (log.)	1.130	1.008	1.268	0.036	1.117	0.995	1.253	0.060	0.954	0.833	1.093	0.499
	Elementary educ.	1				1				1			
	Secondary educ.	1.202	0.929	1.555	0.161	1.264	0.961	1.662	0.093	1.274	0.717	2.264	0.409
	University educ.	1.204	0.862	1.680	0.276	1.129	0.763	1.671	0.545	2.742	1.109	6.778	0.029
	Index lifestyle	1.187	1.105	1.275	0.000	1.280	1.178	1.392	0.000	1.648	1.369	1.983	0.000
	Index psychosocial	1.613	1.416	1.837	0.000	1.719	1.499	1.971	0.000	1.454	1.123	1.882	0.004
	Central-Eastern Europe	1				1				1			
	Western Europe	1.519	1.111	2.077	.009	2.298	1.578	3.347	0.000	2.179	1.053	4.510	0.036
	Southern Europe	1.778	1.325	2.385	.000	1.701	1.197	2.417	0.003	1.311	0.634	2.710	0.465

Source: own calculations

The results of the analysis of healthy ageing predictors across the groups of selected European countries point to the importance of the lifestyle and psychosocial index in all countries (Table 5). In Western Europe the odds of being healthy at old age increase by 34 % with each point of the lifestyle index for males and 35.4 % for females; in Southern Europe by 43.6 % for males and 24.4 % for females and in Central-Eastern Europe by 27.7 % for males and 13.9 % for females. Again, the psychosocial index of social activities, networking and life satisfaction is found to be very important, increasing the likelihood of healthy ageing by 74 % per each index point for males and 68.8 % for females in Western European countries, 68.7 % for males and 63.4 % for females in Southern European countries, and 53 % for males and 52.7 % for females in Central-Eastern European countries.

**Table 5:** Multidimensional analysis of health ageing predictors by country groups – logistic regression results

		Western Europe				Southern Europe				Central-Eastern Europe			
Male or female		OR	95 % CI			OR	95 % CI			OR	95 % CI		
			Lower level	Upper level	*p*		Lower level	Lower level	*p*		Lower level	Lower level	*p*
Male	60–67	1				1				1			
	68–79	0.630	0.478	0.831	0.001	0.840	0.660	1.068	0.155	0.710	0.519	0.973	0.033
	> = 80	0.425	0.288	0.627	0.000	0.466	0.330	0.658	0.000	0.451	0.263	0.773	0.004
	Income (log)	0.980	0.868	1.106	0.741	0.992	0.896	1.099	0.883	1.088	0.858	1.378	0.487
	Elementary educ.	1				1				1			
	Secondary educ.	0.972	0.715	1.321	0.856	1.680	1.213	2.326	0.002	0.929	0.650	1.327	0.684
	University educ.	1.240	0.893	1.723	0.200	2.015	1.290	3.147	0.002	1.251	0.788	1.984	0.342
	Index lifestyle	1.340	1.232	1.457	0.000	1.436	1.336	1.544	0.000	1.277	1.163	1.402	0.000
	Index psychosocial	1.741	1.458	2.079	0.000	1.687	1.494	1.904	0.000	1.530	1.301	1.799	0.000
Female	60–67	1				1				1			
	68–79	0.710	0.662	1.122	0.269	0.478	0.377	0.607	0.000	0.474	0.331	0.679	0.000
	> = 80	0.451	0.403	0.851	0.005	0.248	0.169	0.362	0.000	0.270	0.142	0.514	0.000
	Income (log)	1.260	1.079	1.472	0.003	1.033	0.942	1.133	0.492	0.971	0.819	1.151	0.736
	Elementary educ.	1				1				1			
	Secondary educ.	0.969	0.742	1.266	0.818	1.677	1.188	2.367	0.003	1.473	1.038	2.089	0.030
	University educ.	0.909	0.654	1.263	0.571	1.587	0.968	2.601	0.067	1.930	1.089	3.419	0.024
	Index lifestyle	1.354	1.237	1.482	0.000	1.244	1.147	1.350	0.000	1.139	1.027	1.264	0.014
	Index psychosocial	1.688	1.411	2.020	0.000	1.634	1.443	1.850	0.000	1.527	1.274	1.830	0.000

Source: own calculations

## Discussion

In this study, the relation between healthy ageing (i.e. being healthy at older age) and three groups of factors: lifestyle index, psychosocial index and socio-demographics is analyzed. The definition of healthy ageing used in this study refers to subjective multi-dimensional indicators of being healthy, i.e. self-reported by older people health status, functional abilities and a psychosocial construct like positive meaning of life. Functional ability associated with the level of independence in everyday activity could significantly influence not only self-assessment of health status, but also the chances and abilities to participate in social life, especially out of home activities. It could also influence the general psychological well-being. The last dimension of the definition is strongly related to psychological well-being and social relations in older age.

Differences in the subjective assessment of healthy ageing that are found reflect variations in the health status of older Europeans reported in other studies as well. According to the WHO [76] and Eurostat [77] data life expectancy of older Europeans (65+) is higher in Southern and Western European than in Central-Eastern Europe and reversely the prevalence of chronic conditions is higher in Central-Eastern Europe than in other regions, especially in Western Europe. Objective indicators coming from the epidemiological studies (mortality, LE) well document differences in health status across European countries.

Presented analysis points to the differences in healthy ageing (i.e. being healthy at older age) assessed by a multi-dimensional, subjective measure. Variations in healthy ageing could be attributable to health and social inequalities in older age, but also to factors developed and accumulated throughout lives. Similarly to studies pointing to educational inequalities in health [27, 47], there is an educational gradient in healthy ageing observable in this study as the proportion of individuals being healthy at older age increases with the educational level. Usually the role of education is analyzed in relation to the level and potential for health education, health beliefs, and awareness of the risk factors in several health conditions as well as better understanding of individual susceptibility to specific diseases. This could be also attributed to various factors related to social position, including healthier lifestyle throughout life [45] and better access to information and care. At the population level, the observed differences can be related to historical developments and different life experiences as well as life choices of younger and-especially-older populations between different European regions (i.e. access to education or employment status of men and women). In Poland and Hungary, a lower share of people ageing healthily might be attributable to poor social and economic conditions in their younger years of the communist era and during the economic and political transition period.

The results of the health predictors by age groups confirm that undertaking physical activity, healthy diet based on high consumption of vegetables and fruits, high consumption of liquids and regular meals are crucial and positively related to health outcomes, fitness and well-being in older age, as also reported by others [1]. It is necessary to point to earlier findings of Robinson et al. [40] that healthy lifestyle is important for each age group – even the oldest old. At the same time, presented results confirm earlier findings that participation in social activities and social networking might decrease the risk of morbidity, functional decline and mental illness [59–61] adding that the results are important for all age groups and for both sexes. It should be noted that even after controlling for life-style, social participation and networking, educational gradient in healthy ageing is found for males below the age of 68 with higher probability of healthy ageing for males with a secondary education or an university degree. This is partly in line with earlier findings of various European studies [27, 32] pointing to the differences in health status in older age between educational groups. The positive relation between education and healthy ageing of men might be related to their prolonged outside activity, especially on the labor market, which is more common among people with higher education degree. This relation is not observed however for women. For them it is not education but income that increases the probability of healthy ageing in the group of the youngest old (below the age of 68) while. Such results would suggest that for women, economic standing is more adequate social status indicator than education and could be a better predictor of healthy ageing, this however would require further studies. The relation between healthy ageing and education or income is insignificant for individuals above the age of 68. These might confirm earlier finding that inequalities in health tend to diminish with age [27].

Cross-country analysis points that in countries with higher economic inequalities and higher health inequalities [78] of Southern and Central-Eastern Europe, education plays a role for females as the probability of healthy ageing increases with education, even if lifestyle, psychosocial factors and age are controlled for. In post-communist countries, like Hungary and Poland, this might point to the theory of decomposition of the social status observed in the 1980s where education was found to be more important predictor of social status than income. Still in the studies from 1990s, the correlation between education and income was higher in the Western European countries than in Southern or Central-Eastern European countries [79]. For the older population, the decomposition might have led to differences in social status and health behaviors throughout life that result in better propensity for healthy ageing in people with secondary and higher education, and in greater importance of education than income with respect to health outcomes and well-being. In Western European countries, education is not among the important predictors of healthy ageing, but income is still found to be significantly related to health. These results might partly point to the existence of socio-economic inequalities in health among older people [27], but rather among females than males. The differences between men and women would need further studies.

Overall, it should be noted that while differentiating between age groups and countries, the study points to the homogeneity of positive correlates of healthy ageing (i.e. being healthy at older age) with healthy lifestyle characterized by undertaking physical activity, adequate nutrition and non-smoking as well as social activities, including leisure and social networking. The psycho-social characteristics related to participation in outside activities, being rooted in social networks and life satisfaction are of similar importance for the health status as undertaking healthy behaviors by older people. The study adds to the previous research that these correlates are important in all age groups, and points that even the oldest old might benefit from the healthy lifestyle and social participation and networking. From the public health policy point of view, it is an important finding that investments in healthy diet and physical activity as well as stimulation of social networks and activities are related to positive self-perception of health status, high level of functional abilities and perceived meaning of life in older age, even for the oldest old. The study also points that in order to increase the healthy ageing potential in the European countries (and increase healthy life expectancy by 2 years, what is a public health goal for 2020), public health policy should also aim at investments in the health promotion programs and initiatives targeted to the older population, stimulating physical health via adequate nutrition in line with the WHO recommendations, physical exercises and non-smoking policies, accompanied by stimulation of social networks and active ageing measures oriented at social participation.

The strength of the presented study is in assessing healthy ageing predictors for several countries characterized not only by different cultural and socio-economic environment, but also by different social positions of older people in society and variations in policy measures addressing health. In presented countries, social policy focused on the health status of older people varies due to differences in priorities, economic and social resources involved as well as management. According to Raphael [80], countries of continental and southern Europe less frequently directly address public health issues, incorporating health measures into general social policies, than Anglo-Saxon countries. This is even less frequent in Central-Eastern European countries where the awareness of the social and health policy towards ageing is only raising. Thus, results of presented analyses can be used to improve social and public health policy depending on the country-specific circumstances.

The study however has some limitations related to the definition of healthy ageing. Presented definition is based on the three individual level indicators, however the authors are aware that there are various definitions of healthy status in older age and healthy aging might be adopted. The selection of the three domains was based on the literature review and the database review. The literature review pointed to numerous definitions of healthy ageing [2], which are briefly discussed above with the three element: self-assessed heath, functional abilities and psychological well-being or capabilities present in many of them. The definition adopted in this study is found by the authors as the best suited definition representing all three domains in the SHARE study. The SHARE survey of the years 2010–2011 was selected as the best database providing individual level information on health status in the three domains identified, life-style patterns, social networks and activity as well as demographic and socio-economic information. Other available databases (with public access) included specific dimensions of health, however with narrower scope (European Social Survey, International Social Survey Programme, etc.). The study is of preliminary character, pointing to the need for further studies that would allow to identify more country specific features of healthy ageing, using country specific definitions that could be addressed by policies at the national level. Furthermore, a dynamic approach to the analysis of healthy aging could be adopted in future research.

## Conclusions

Presented study identifies the set of predictors related to healthy ageing (i.e. being healthy at older ager) as defined by three domains: good health status self-assessment, functional abilities and perception of meaning in life. The study allows for identification and discussion of factors that are of importance for well-defined and targeted public health policy, and especially health promotion programs oriented towards healthy ageing.

Policy conclusions that can be drawn from the research point that the main concern for public health policy oriented towards health and high quality of life in older age include stimulation of healthy lifestyle characterized by vigorous or moderate physical activity of older people, high consumption of vegetables and fruits, regular nutrition and high consumption of liquids. These behavioral patterns are found to be positively related in all countries covered with the study, for men and women and for all age groups. Importantly, even for the oldest age group (80+), the healthy lifestyle is beneficial. The patterns identified are similar for men and women.

Whilst social participation and social networking are not within the scope of public health actions, they are also of importance for healthy ageing. Social participation, as defined in the study includes labor market participation of the labor market active age people as well as involvement in outdoor and indoor leisure activities, which older people might engage in. Next to social participation, also social networking and satisfaction are important predictors of healthy ageing because they facilitate the feeling of belonging and being a part of society, and significantly influence the social integration of older people together with younger generations. The meaning of life, strongly associated with the will to live, usually is created by positive emotions and present and future life plans. It might be important to encourage older people to participate and network also via the health promotion tools.

The idea of health promotion among older people has been developed relatively recently in European countries, especially in the Central-Eastern Europe. There had been a long-lasting belief that supporting health promotion in younger generations will result in improved health outcomes measured by a decrease in morbidity and mortality, and improvement in health-related quality of life. Presented data show that healthy lifestyle and satisfactory psychosocial functioning are significantly related to better quality of life in older age, even above 80 years of age. Challenges in front of the health promoters include achieving better results in healthy lifestyle promotion among older people and developing positive attitudes towards health promotion in older age by showing benefits of healthy ageing.

## Abbreviations

ADL, activities of daily living; SAH, self assessed health; SHARE, Survey of Health Ageing and Retirement in Europe; WHO, World Health Organization
